# Neoadjuvant Chemotherapy of Patients with Early Breast Cancer Is Associated with Increased Detection of Disseminated Tumor Cells in the Bone Marrow

**DOI:** 10.3390/cancers14030635

**Published:** 2022-01-27

**Authors:** Léa Volmer, André Koch, Sabine Matovina, Dominik Dannehl, Martin Weiss, Ganna Welker, Markus Hahn, Tobias Engler, Markus Wallwiener, Christina Barbara Walter, Ernst Oberlechner, Sara Yvonne Brucker, Klaus Pantel, Andreas Hartkopf

**Affiliations:** 1 Department of Women’s Health, University Medical Center Tübingen, 72076 Tübingen, Germany; Sabine.Matovina@med.uni-tuebingen.de (S.M.); Dominik.Dannehl@med.uni-tuebingen.de (D.D.); Martin.Weiss@med.uni-tuebingen.de (M.W.); markus.hahn@med.uni-tuebingen.de (M.H.); tobias.engler@med.uni-tuebingen.de (T.E.); Christina-Barbara.Walter@med.uni-tuebingen.de (C.B.W.); ernst.oberlechner@med.uni-tuebingen.de (E.O.); sara.brucker@med.uni-tuebingen.de (S.Y.B.); Andreas.hartkopf@med.uni-tuebingen.de (A.H.); 2 Research Institute for Women’s Health, University Medical Center Tübingen, 72076 Tübingen, Germany; Andre.Koch@med.uni-tuebingen.de (A.K.); Ganna.Welker@med.uni-tuebingen.de (G.W.); 3 Department of Gynecology and Obstetrics, University Medical Center Heidelberg, 69120 Heidelberg, Germany; markus.wallwiener@med.uni-heidelberg.de; 4 Department of Tumor Biology, University Medical Center Hamburg-Eppendorf, 20246 Hamburg, Germany; pantel@uke.de

**Keywords:** breast cancer, disseminated tumor cells, neoadjuvant chemotherapy, circulating tumor cells

## Abstract

**Simple Summary:**

Disseminated tumor cells (DTCs) present in the bone marrow of breast cancer patients are an indicator of minimal residual disease and micrometastatic spread. These cells can already be found at the earliest disease stages and are associated with poorer outcomes. In preclinical models, neoadjuvant chemotherapy was shown to promote micrometastatic spread. The aim of this large single-center retrospective study was to compare the frequency and prognostic significance of DTC detection between patients treated with neoadjuvant chemotherapy and treatment-naive patients.

**Abstract:**

Preclinical data suggest that neoadjuvant chemotherapy (NAT) may promote micrometastatic spread. We aimed to compare the detection rate and prognostic relevance of disseminated tumor cells (DTCs) from the bone marrow (BM) of patients with early-stage breast cancer (EBC) after NAT with that of therapy-naive EBC patients. DTCs were identified from BM samples, collected during primary surgery. Patients who received NAT were compared to patients who received chemotherapy after surgery. In total, 809 patients were analyzed. After NAT, 45.4% of patients were DTC-positive as compared to 19.9% of patients in the adjuvant chemotherapy group (*p* < 0.001). When sampled in patients who had undergone NAT, the detection of DTCs in the BM was significantly increased (OR: 3.1; 95% confidence interval (CI): 2.1–4.4; *p* < 0.001). After NAT, DTC-positive patients with ≥2 DTCs per 1.5 × 10^6^ mononuclear cells in their BM had an impaired disease-free survival (HR: 4.8, 95% CI: 0.9–26.6; *p* = 0.050) and overall survival (HR: 4.2; 95% CI: 1.4–12.7; *p* = 0.005). The higher rate of DTC-positive patients after NAT as compared to a treatment-naive comparable control cohort suggests that NAT supports tumor cell dissemination into the bone marrow. DTC positivity in BM predicted relapse in various distant organs, implying that tumor cell dissemination was not restricted to the bone marrow.

## 1. Introduction

Breast cancer is the most common type of cancer in women in the western world. Despite modern treatment, the disease may recur at distant sites even in patients without lymph node involvement and small tumors that have been completely removed. This implies that the disease spreads early and remains in a dormant state, a phenomenon called minimal residual disease (MRD) [1]. As a surrogate of MRD, disseminated tumor cells (DTC) can be detected in the bone marrow (BM) of 20–30% of patients with early breast cancer (EBC) and their detection is associated both with worse outcomes and with locoregional and distant recurrence [2,3,4].

The aim of systemic treatment in EBC is to eradicate MRD. Currently, neoadjuvant chemotherapy (NAT) is increasingly being used because it allows for the monitoring of treatment response, which can be assessed by pathologic complete response (pCR) or scoring systems such as the CPS + EG score [5]. While pCR is usually defined as the absence of any invasive tumor residuals in the breast or lymph nodes following NAT, scoring systems such as the CPS + EG score (clinical-pathologic Scoring System incorporating estrogen receptor-negative disease and nuclear grade 3 tumor pathology) use a pre-treatment clinical stage as well as post-NAT pathologic stage, nuclear grade, and the estrogen receptor status to estimate prognosis [6]. Achieving pCR after NAT is associated with favorable prognosis, yet even patients who achieve a pCR may relapse. Therefore, the monitoring of MRD might help to improve risk stratification after NAT [7].

Interestingly, earlier trials have found relatively high numbers of DTC-positive patients after NAT, while no significant correlation between DTC detection and response to NAT was found [8,9,10]. Moreover, studies with pre-clinical mouse models reported that NAT is associated with tumor propagation and micrometastasis [11]. However, the clinical significance of these findings is unclear, since NAT, as compared to adjuvant chemotherapy, does not appear to increase the risk of distant recurrence [12].

We therefore aimed to compare the proportion and prognostic relevance of DTC positivity when BM was sampled after NAT with the proportion and prognostic relevance of DTC positivity when BM was sampled before adjuvant chemotherapy. In patients who received NAT, we investigated the association between DTC detection and treatment response as determined by pCR and the CPS + EG score.

## 2. Materials and Methods

### 2.1. Study Population

Patients treated with at least four cycles of chemotherapy for EBC (T1-4, N0-3) who underwent surgery at the Department of Women’s Health, University of Tuebingen, Germany, between January 2014 and December 2019, were eligible for this retrospective analysis. Exclusion criteria were recurrent or distant metastatic disease, bilateral breast cancer or a previous history of secondary malignancy. Two treatment groups were defined: in the neoadjuvant group, patients received chemotherapy before surgery, and in the adjuvant group, patients received chemotherapy after surgery. All patients provided written informed consent. The analysis was approved by the ethics committee of Tuebingen University (reference number: 528/2019BO2).

### 2.2. DTC Detection

BM sampling was performed during surgery. Written consent for BM sampling, as well as BM and data processing, was given prior to operation. Hence, in the neoadjuvant group, BM was sampled after chemotherapy, and in the adjuvant group, BM was sampled before chemotherapy. All BM samples were processed within 24 h. Mononuclear cells from the bone marrow were isolated, then spun down onto a glass slide. The presence of DTC (DTC status) was determined by immunostaining against pancytokeratin and cytomorphology (see Appendix A). DTC positivity was defined as at least one pancytokeratin-positive cell with typical cell morphology [13] per 1.5 × 10^6^ cells.

### 2.3. Systemic Treatment

Neoadjuvant or adjuvant chemotherapy was applied according to national treatment guidelines [14,15]. In the adjuvant group, tumor stage was routinely determined by pathological examination based on the excised tumor at the time of surgery. In the neoadjuvant group, tumor size and nodal status were determined by clinical examination and imaging modalities before the first treatment cycle. In the neoadjuvant group, the CPS + EG score was calculated according to Jeruss et al. and pCR was defined as ypT0/ypTis and ypN0 [6,7].

### 2.4. Statistical Analysis

Correlations between DTC status and a patient’s characteristics were evaluated using the chi-square test. Factors promoting tumor cell dissemination were assessed by using a multivariate logistic regression. Factors that achieved statistical significance at *p* < 0.1 in the univariate analysis for DTC positivity were considered for multivariate analysis. Odds ratios (OR) and confidence intervals (CI) were calculated. For survival analysis, duration from BM aspiration to any distant or locoregional disease recurrence (disease-free survival, DFS) and death of any cause (overall survival, OS) were calculated separately. If no event occurred, data were censored at the time of last follow-up. Kaplan–Meier curves were plotted and compared using the log-rank test. For BM samples after NAT, we furthermore analyzed whether higher numbers of DTCs impact DFS and OS. For this purpose, two groups were defined: patients with 0–1 DTC per 1.5 × 10^6^ mononuclear cells and patients with ≥2 DTCs per 1.5 × 10^6^ mononuclear cells. The median follow-up was calculated with the reverse Kaplan–Meier method. All statistical analyses were performed using JMP15 (SAS^®^). Significance level was set at *p* < 0.053.

## 3. Results

### 3.1. Patient’s Characteristics

In total, 809 patients were included in our retrospective analysis. The median age at initial diagnosis was 53 years. BM was sampled after NAT (neoadjuvant group) in 207 (25.6%) and before adjuvant chemotherapy (adjuvant group) in 602 (74.4%) patients. As displayed in Table 1, most tumors were of no special type (87.8%) and T2–4 (65.3%). Axillary lymph node involvement was found in 400 (49.9%) patients. Tumors were luminal-like (i.e., hormonal receptor-positive and HER2-negative) in 426 (53.1%), HER2-positive in 215 (26.8%) and triple-negative in 161 (20.1%) patients. Patients in the neoadjuvant group were more often premenopausal (57.0% vs. 42.3%, *p* < 0.001) and had a higher proportion of G3 (66.0% vs. 53.6%, *p* = 0.002) as well as triple-negative (26.2% vs. 18.0%, *p* < 0.001) or HER2-positive (35.4% vs. 23.8%, *p* < 0.001) tumors. Moreover, the initial tumor size was greater (87.0% vs. 57.9% of initial T2-4, *p* < 0.001) and axillary lymph nodes were more often positive (70.0% vs. 42.8%, *p* < 0.001) than in the adjuvant group. When treated with NAT, pCR was achieved in 82 (39.6%) cases and the median CPS + EG was five.

### 3.2. Detection of Disseminated Tumor Cells

Overall, 214 (26.5%) of all patients were DTC-positive (Table 2). In the neoadjuvant group, a significantly higher proportion of patients were DTC-positive than in the adjuvant group (94/207, 45.4% vs. 120/602, 19.9%, *p* < 0.001). Patients showing tumor cell dissemination into their BM had larger tumors (*p* = 0.006), and lymph node invasion was observed more frequently (*p* = 0.001). Figure A2 shows the number of DTCs that were detected in patients after NAT and before adjuvant chemotherapy, respectively. As most DTC-positive patients harbored 1–2 DTCs/1.5 × 10^6^ mononuclear cells in their BM, we defined another cut-off for DTC detection and found 24 (11.6%) patients from the neoadjuvant group, and in 10 (1.7%) of the patients from the adjuvant group (*p* < 0.001) with at least 2 DTCs/1.5 × 10^6^ mononuclear cells.

Table 3 shows the proportion of DTC detection in the neoadjuvant treatment group according to the patients’ characteristics. Menopausal status, age, histological type or subtype, initial tumor size and lymph node involvement did not differ between DTC-positive and DTC-negative patients. A significantly higher proportion of patients with a histological type other than a non-special type had higher DTC counts (≥2 DTCs/1.5 × 10^6^ cells) in their BM (27.8% vs. 10.1%, *p* = 0.047). Moreover, the pCR rates were similar between patients with or without tumor cell dissemination into their bone marrow (34.0% in DTC-positive vs. 44.2% in DTC-negative patients, *p* = 0.134). However, the CPS + EG score was more often >4 when DTC were detected (45.5% vs. 36.4%, *p* = 0.036). Patient characteristics according to the DTC detection in patients in the adjuvant group are displayed in Table A1.

In the multivariate analysis (Table 4), the time point of BM collection, i.e., before (neoadjuvant group) or after systemic therapy (adjuvant group), was found to be the strongest factor for DTC detection (odds ratio: 3.1; 95% CI: 2.1–4.4; *p* < 0.001).

### 3.3. Survival Analysis

**Figure 1:**
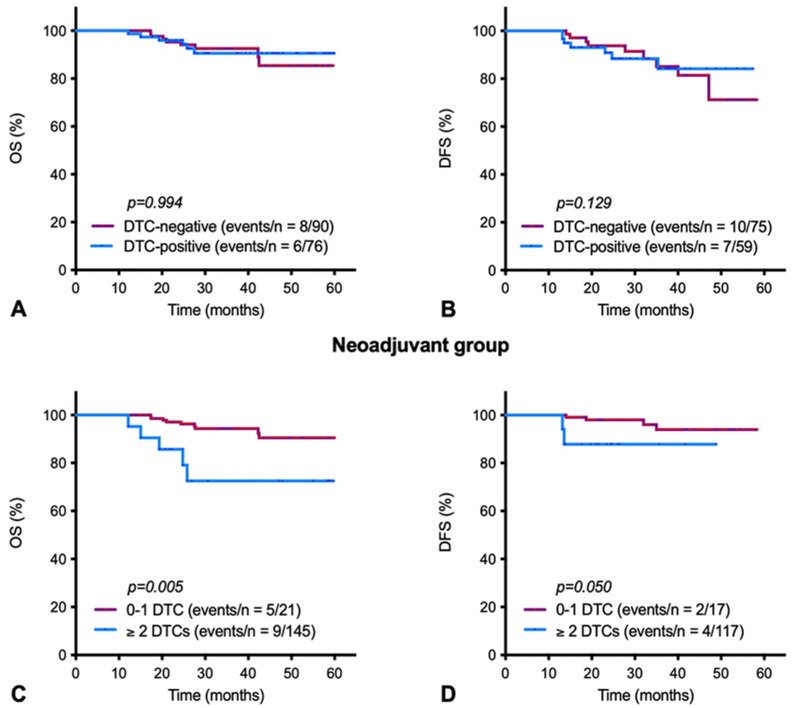
Univariate survival analysis by disseminated tumor cell (DTC) status after neoadjuvant chemotherapy. Kaplan–Maier plots of (**A**,**C**) overall survival (OS) and (**B**,**D**) disease-free survival (DFS) for DTC detection in bone marrow samples of patients who had received neoadjuvant chemotherapy. (**A**,**B**) DTC-negative patients (purple line) as compared to DTC-positive patients and (**C**,**D**) patients with 0-1 DTCs per 1.5 × 10^6^ mononuclear cells (purple line) as compared to patients with ≥2 DTCs/1.5 × 10^6^ mononuclear cells (blue line).

**Figure A1:**
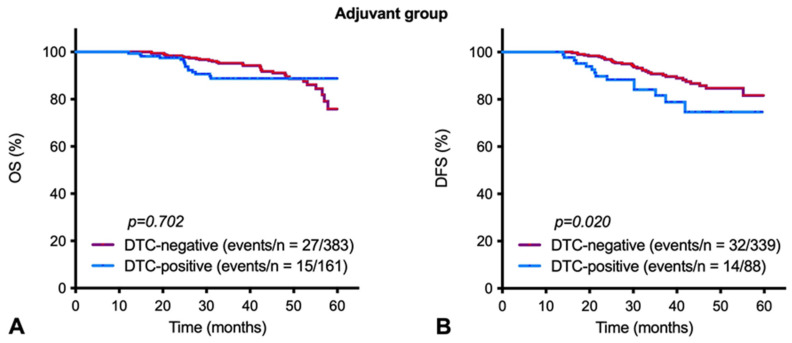
Univariate survival analysis by disseminated tumor cell (DTC) status before the start of adjuvant chemotherapy. Kaplan–Maier plots of (**A**) overall survival (OS) and (**B**) disease-free survival (DFS) for DTC-negative (purple line) as compared to DTC-positive (blue line) treatment-naïve patients.

**Figure A2:**
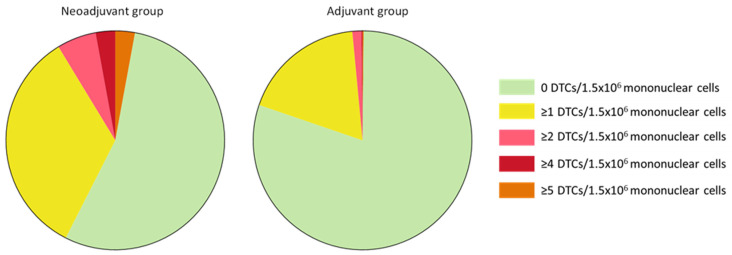
Quantification of disseminated tumor cells (DTCs) in patients with bone marrow aspiration before (**left**) and after chemotherapy (**right**). Samples were classified as DTC-negative (0 DTCs/1.5 × 10^6^ mononuclear cells) or DTC-positive (≥1 DTC/1.5 × 10^6^ mononuclear cells OR ≥ 2 DTCs/1.5 × 10^6^ mononuclear cells OR ≥ 4 DTCs/1.5 × 10^6^ mononuclear cells OR ≥ 5 DTCs/1.5 × 10^6^ mononuclear cells).

The median follow-up was 45.1 months for OS and 32.3 months for DFS. Follow-up data were available for 134 (DFS) and 166 (OS) of patients in the neoadjuvant group. In the neoadjuvant group (Figure 1A,B), there was no significant effect of DTC positivity on OS (hazard ratio (HR): 1.1 95% confidence interval (CI): 0.4–2.7; *p* = 0.994) or DFS (HR 1.4; 95% CI: 0.6–3.4; *p* = 0.129). However, in patients who harbored higher numbers of DTCs in their BM (≥2 DTCs/1.5 × 10^6^ mononuclear cells), we found a significantly lower OS (HR: 4.2; 95% CI: 1.4–12.7; *p* = 0.005) and DFS (HR: 4.8, 95% CI: 0.9–26.6; *p* = 0.050) than in patients with <2 DTCs/1.5 × 10^6^ mononuclear cells (Figure 1C,D). In the adjuvant group (Figure A1), DTC positivity was significantly associated with worse DFS (HR: 2.2; 95% CI: 1.0–4.6; *p* = 0.043), whereas no significant association was found with OS (HR: 1.9; 95% CI: 0.9–3.9; *p* = 0.702). Due to the very low number of patients with ≥2 DTCs/1.5 × 10^6^ mononuclear cells in the adjuvant group, we did not perform a survival analysis for this threshold. The sites of recurrence are displayed in Table A2 and Table A3. There was no association between DTC positivity and the location of metastases at first diagnosis of distant relapse (bone-only vs. other sites).

## 4. Discussion

Several reports have found that chemotherapy in the presence of a primary tumor might induce tumor cell extravasation and intravasation to metastatic sites [11,16,17,18,19,20]. We therefore investigated tumor cell dissemination into the BM after completion of NAT and compared the rate of DTC-positive patients in this group with that of treatment-naive patients. We found that tumor cell dissemination is highly increased in patients with EBC who have received systemic therapy before definitive surgery of the primary tumor.

To minimize a potential bias from the selection of patients for NAT, only patients who received chemotherapy were included in the comparative adjuvant group. Hence, a markedly high-risk population was studied (most patients were nodal-positive, had G3 tumors, or a non-luminal subtype). Nevertheless, patients who received NAT as compared to adjuvant chemotherapy were younger, had higher tumor stages and a more aggressive tumor biology (triple-negative or HER2-positive). To address these limitations, we performed a multivariate regression analysis and found the timepoint of chemotherapy to be the strongest independent factor of DTC positivity. Importantly, the rate of DTC-positive patients among treatment-naive patients (adjuvant group) was in line with detection rates in earlier trials [21,22,23,24]. It is thus unlikely that the increased rate of DTC-positive patients in the neoadjuvant group is only due to different patient characteristics as compared to the adjuvant group.

Several studies in mouse mammary tumors have highlighted how cytotoxic agents contribute to the development of metastases. For example, the chemotherapy-induced expression of VEGFR-1 on endothelial cells can create an environment conducive to tumor cell homing [17]. In addition, the density and activity of cancer cell invasion sites are increased in residual tumors of patients treated with NAT, which may increase the risk of tumor cell dissemination [25]. Furthermore, neoadjuvant chemotherapy increases the release of tumor-derived extracellular vesicles that might facilitate the formation of metastasis [11,26]. Moreover, several studies have highlighted the potentially pro-tumorigenic effects of chemotherapy both directly in cancer cells [27] and in the tumor microenvironment [28]. These effects often correlate with a decreased rate of DFS and increased recurrence rates. Although we and others could not find a significant association between DTC detection and pCR, patients with a high CPS + EG score were more likely to harbor DTC in BM, which is in line with data from Magbanua et al., who found that the DTC status after NAT correlates with residual cancer burden (RCB) [10,29,30,31]. The fact that the CPS + EG score depends not only on treatment response but also on tumor biology (grading and ER status) might, however, explain our observation as well [6].

Patients with residual disease after NAT have an impaired prognosis after NAT. Although recent meta-analyses have shown that there is no difference in terms of prognosis whether chemotherapy is given before or after surgery [12], it cannot be excluded that the marked differences in metastatic-free survival between patients with and without pCR are, at least in part, due to a pro-metastatic effect in those patients who do not respond to NAT. In the current study, we found no association between survival and the DTC status after NAT, consistent with some but not all previous findings [10,29]. This finding contrasts with the worsened DFS in DTC-positive patients in the adjuvant group, i.e., when BM sampling was performed before chemotherapy. The increased detection rate of DTC after NAT suggests that a large proportion of DTC will never develop into manifest metastases. Indeed, only when we looked at patients with higher numbers of DTCs in their bone marrow (≥2 DTCs/1.5 × 10^6^ mononuclear cells) after the completing NAT were we able to confirm a poorer prognosis in the neoadjuvant group too (Figure 1C,D). Importantly, relapse after DTC detection was not restricted to the bone as the first site of metastasis, suggesting that tumor cells not only disseminate into the bone marrow but also to distant viscera. Further characterization of DTC could help identify those DTCs with high metastatic potential. Interestingly, TWIST1, a transcription factor that plays a pivotal role in metastasis by promoting epithelial-mesenchymal transition (EMT) was part of the gene expression signature previously identified in DTC of breast cancer patients [32]. TWIST1 expression was associated with the occurrence of distant metastasis even in BM samples of patients that have received NAT, supporting the view that tumor cells undergoing EMT might have higher resistance to chemotherapy [33]. Moreover, DTCs detected after NAT may have been apoptotic due to cytotoxic treatment, which may also explain the different impacts of DTCs on survival between treated and treatment-naïve patients. Higher patient numbers and longer follow-up may confirm worsened survival already with fewer DTCs in the NAT group.

Besides the retrospective character of our study and the lack of randomization into treatment groups (neoadjuvant and adjuvant, respectively), a major limitation of the current analysis is that it cannot be determined whether the timing of BM sampling (before or after chemotherapy) or the timing of chemotherapy (neoadjuvant versus adjuvant) is responsible for the high detection rate in the neoadjuvant group. For example, most patients treated with NAT had received granulocyte-colony stimulating factors (G-CSF) during chemotherapy, which leads to higher amounts of mononuclear cells and might therefore lead to false positive DTC detection. Synnestvedt et al. performed BM sampling after six cycles of adjuvant chemotherapy and detected DTC only in 8.7% of the patients [34]. This suggests that the higher proportion of DTC-positive patients can be attributed to the timepoint of chemotherapy, i.e., the neoadjuvant administration. Repeated BM sampling of the same patient (before and after neoadjuvant chemotherapy) would be optimal to show that the number of DTCs in the BM increases during NAT. However, this was not performed due to the increased morbidity of repeated BM sampling and the burden of BM sampling without general anesthesia. In a substudy of the Neotax trial, Mathiesen et al. investigated DTC status in 66 patients with stage III/IV breast cancer before (BM1) and after neoadjuvant chemotherapy (BM2). The authors found no significant association between DTC detection at the BM1 and BM2. However, in contrast to our results, the DTC detection rate was not increased after NAT, possibly due to the lower chemotherapy dosage in the Neotax study compared with our cohort [29]. To avoid morbidity related to BM sampling circulating tumor cells (CTCs) can also be detected in the peripheral blood. In a large meta-analysis evaluating circulating tumor cell (CTC) detection from the peripheral blood before and after NAT, Bidard et al. did not find CTC counts to be increased after NAT [35]. However, DTC and CTC detection in early breast cancer are not related to each other, probably due to the lower sensitivity of CTC detection in early-stage breast cancer and to the shorter half-life of CTCs [36]. Of note, Konig et al. found an inverse association between CTC detection and the formation of tumor-derived extracellular vesicles during NAT, however, the reason for this observation remains unclear [26].

Currently, no implications on clinical routine can be drawn from our results. Further clinical studies, which in addition to the pure detection of DTCs also include their characterization, are necessary to identify DTCs with a high metastatic potential and ideally to treat them with targeted drugs. To monitor MRD, further methods of liquid biopsy, such as the deletion and characterization of CTCs or circulating tumor DNA (ctDNA), should be investigated.

## 5. Conclusions

In conclusion, the rate of DTCs in the BM of patients after NAT was higher than in a comparable control cohort who received adjuvant chemotherapy. This suggests that NAT supports tumor cell dissemination into the bone marrow. Detection of DTCs was not associated with therapy response, suggesting that single tumor cells may survive NAT even in cases of a pCR. Patients who harbored higher numbers of DTCs in their BM after NAT were at an increased risk of distant relapse or death. As these relapses occurred at various sites, NAT might increase tumor cell dissemination not only into the BM but also into other organs.

## Figures and Tables

**Table 1:** Characteristics of patients that received neoadjuvant as compared to adjuvant chemotherapy.

	All Patients	Neoadjuvant Group	Adjuvant Group	*p*-Value *
All patients, *n*	809	207	602	
Mean age (years)	54.0	50.5	55.4	<0.001
Menopausal status, *n* (%)				<0.001
premenopausal	371 (46.1)	118 (57.0)	253 (42.3)	
postmenopausal	434 (53.9)	89 (43.0)	345 (57.7)	
Histology, *n* (%)				0.063
no special type	710 (87.8)	189 (91.3)	521 (86.5)	
other subtypes	99 (12.2)	18 (8.7)	81 (13.5)	
Nuclear grade, *n* (%)				0.002
G1–2	348 (43.2)	70 (34.0)	278 (46.4)	
G3	457 (56.8)	136 (66.0)	321 (53.6)	
Initial tumor size, *n* (%) **				<0.001
T1	280 (34.7)	27 (13.0)	253 (42.1)	
T2–4	528 (65.3)	180 (87.0)	348 (57.9)	
Initial nodal status, *n* (%) **				<0.001
N0	402 (50.1)	62 (30.0)	340 (57.2)	
N1–3	400 (49.9)	145 (70.0)	255 (42.8)	
Subtype, *n* (%)				<0.001
triple-negative	161 (20.1)	54 (26.2)	107 (18.0)	
luminal-like ***	426 (53.1)	79 (38.3)	347 (58.2)	
HER2-positive	215 (26.8)	73 (35.4)	142 (23.8)	
pCR ****				
yes	-	82 (39.6)	-	-
no	-	125 (60.4)	-	-
CPS + EG score ****				
CPS + EG score ≤ 4	-	77 (41.2)	-	-
CPS + EG score > 4	-	110 (58.8)	-	-

* chi^2^ test was used for categorical variables; *t*-test was used for continuous variables (age). ** Tumor size was assessed before the start of systemic therapy (clinically before neoadjuvant and histologically before adjuvant chemotherapy). *** Luminal-like is defined as hormonal receptor-positive/HER2-negative. **** pCR and CPS + EG score were determined for patients with NAT only.

**Table 2:** Patient characteristics by disseminated tumor cell (DTC) status.

	All Patients	DTC-Positive *		≥2 DTCs/1.5 × 10^6^ Cells *	
	*n*	*n* (%)	chi^2^ *p*-Value	*n* (%)	chi^2^ *p*-Value
Total	809	214 (26.5)		34 (4.2)	
Treatment Group *					
Neoadjuvant group	207	94 (45.4)		24 (11.6)	
Adjuvant group	602	120 (19.9)	<0.001	10 (1.7)	<0.001
Menopausal status			0.539		
premenopausal	371	102 (27.5)		18 (4.9)	
postmenopausal	434	111 (25.6)		16 (3.7)	0.414
Histology			0.843		
non-special type	710	187 (26.3)		27 (3.8)	
other subtypes	99	27 (27.3)		7 (7.1)	0.159
Nuclear grade			0.252		
G1–2	348	85 (24.4)		13 (3.7)	
G3	457	128 (28.0)		21 (4.6)	0.546
Initial tumor size **			0.006		
T1	280	58 (20.7)		2 (3.6)	
T2–4	528	156 (29.6)		24 (9.5)	0.122
Initial nodal status **			0.001		
N0	403	86 (21.3)		11 (2.7)	
N1–3	400	126 (31.5)		23 (5.8)	0.032
Subtype			0.579		0.166
Triple-negative	44	14 (31.8)		11 (6.8)	
Luminal-like ***	430	113 (26.3)		17 (4.0)	
HER2-positive	211	53 (25.1)		6 (2.8)	

* In the neoadjuvant group, bone marrow was sampled after neoadjuvant chemotherapy; in the adjuvant group, bone marrow was sampled before adjuvant chemotherapy. ** Tumor size was assessed before the start of systemic therapy (clinically before neoadjuvant and histologically before adjuvant chemotherapy). *** Luminal-like is defined as hormonal receptor-positive/HER2-negative. DTC = disseminated tumor cells.

**Table 3:** Patient characteristics according to the detection of disseminated tumor cells (DTCs) in patients who received neoadjuvant chemotherapy.

	Total	DTC-Positive *n* (%)	*p*-Value	≥2 DTCs/1.5 × 10^6^ Cells *n* (%)	chi^2^ *p*-Value
All patients	207	94 (45.4)		24 (11.6)	
Menopausal status					
premenopausal	118	53 (44.9)		14 (11.9)	
postmenopausal	89	41 (46.1)	0.869	19 (11.2)	0.889
Histology					
non-special type	189	85 (45.0)		19 (10.1)	
other subtypes	18	9 (50.0)	0.682	5 (27.8)	0.047
Nuclear grade					
G1–2	70	28 (40.0)		8 (11.4)	
G3	136	66 (48.5)	0.243	16 (11.8)	0.943
Initial tumor size *					
Tis-1	27	9 (33.3)		1 (3.7)	
T2-4	180	85 (47.2)	0.172	23 (12.8)	0.121
Initial nodal status *					
N0	62	26 (41.9)		7 (11.3)	
N1–3	145	68 (46.9)	0.511	17 (11.7)	0.929
Subtype **					
triple-negative	54	28 (51.9)		9 (16.7)	
luminal-like	79	40 (50.6)		10 (12.7)	
HER2-positive	73	26 (35.6)	0.098	5 (6.9)	0.208
pCR					
yes	82	32 (39.0)		6 (7.3)	
no	125	62 (49.6)	0.134	18 (14.4)	0.110
CPS + EG score					
CPS + EG score ≤ 4	77	28 (36.4)		5 (6.5)	
CPS + EG score > 4	110	57 (45.5)	0.036	18 (16.4)	0.036

* Tumor size was assessed before the start of systemic therapy. ** Luminal-like is defined as hormonal receptor-positive/HER2-negative. DTC = disseminated tumor cells.

**Table 4:** Nominal logistic regression of factors influencing disseminated tumor cell (DTC) detection.

Parameter	OR for DTC Detection	95% CI	chi^2^ *p*-Value
Treatment Group *			
Adjuvant group	1.0		
Neoadjuvant group	3.1	2.1–4.4	<0.001
Initial tumor size **			
Tis-1	1.0		
T2–4	1.1	0.8–1.6	0.612
Initial nodal status **			
N0	1.0		
N1–3	1.3	0.9–1.8	0.145

* In the neoadjuvant group, bone marrow was sampled after neoadjuvant chemotherapy; in the adjuvant group, bone marrow was sampled before adjuvant chemotherapy. ** Tumor size was assessed before the start of systemic therapy (clinically before neoadjuvant and histologically before adjuvant chemotherapy). OR = odds ratio; CI = confidence interval; DTC = disseminated tumor cells.

**Figure A3:**
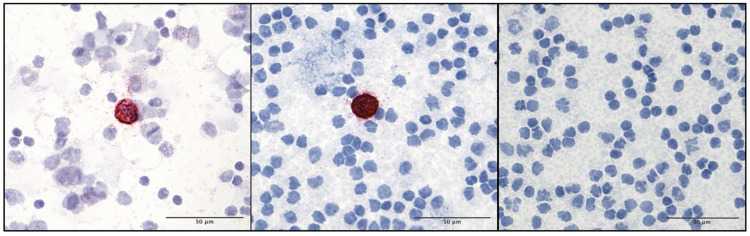
Representative images of: DTC from a patient’s sample (**left**), positive control with an SKBR-3 cell (**middle**), negative control (**right**); taken at 20× magnification.

## Data Availability

The data presented in this study are available on request from the corresponding author.

## Appendix A

Bone marrow processing: Mononuclear cells from the bone marrow were isolated by density centrifugation (Ficoll, 1.077 g/mL, Biochrom, Berlin, Germany). These cells were then spun down onto a glass slide (cytocentrifuge, Hettich, Tuttlingen, Germany) and fixed in 4% formalin. The obtained cytospins were stained using the DAKO Autostainer (DAKO, Glostrup, Denmark). Mouse monoclonal antibodies A45-B/B3 directed against pancytokeratin (Micromet, Munich, Germany) and Keratin 8/18 Ab-1 (Thermo Fisher Scientific, Fremont, USA) were used. For cytokeratin staining, two slides with 1.5 × 10^6^ cells per patient were evaluated, according to the consensus recommendations for standardized tumor cell detection [13]. Each batch of samples was analyzed together with leukocytes from healthy volunteers as negative controls and the human breast cancer cell lines MCF 7 and SKBR 3 as positive controls. Figure A3 shows representative images of each patient’s sample, a positive and negative control.

**Table A1:** Patient characteristics according to the detection of disseminated tumor cells in patients who received adjuvant chemotherapy.

	Total	DTC-Positive *n* (%)	*p*-Value	≥2 DTCs/1.5 × 10^6^ Cells *n* (%)	*p*-Value
All patients	602	120 (19.9)		10 (1.7)	
Menopausal status					
premenopausal	253	49 (19.4)		4 (1.6)	
postmenopausal	345	70 (20.3)	0.780	6 (1.7)	0.881
Histology					
non-special type	521	102 (19.6)		8 (1.5)	
other subtypes	81	18 (22.2)	0.534	2 (2.5)	0.564
Nuclear grade					
G1-2	278	57 20.5)		5 (1.8)	
G3	321	62 (19.3)	0.716	5 (1.6)	0.819
Initial tumor size *					
T1	253	49 (19.4)		1 (3.6)	
T2-4	348	71 (20.4)	0.754	1 (1.4)	0.501
Initial nodal status *					
N0	340	49 (14.4)		4 (1.2)	
N1-3	255	70 (27.5)	0.562	6 (2.4)	0.272
Subtype **					
triple-negative	107	20 (18.7)		2 (1.9)	
luminal-like	347	72 (20.8)		7 (2.0)	
HER2-positive	142	28 (19.7)	0.888	1 (0.7)	0.524

* Tumor size was assessed before the start of systemic therapy (histologically before adjuvant chemotherapy). ** Luminal-like is defined as hormonal receptor-positive/HER2-negative. DTC = disseminated tumor cells.

**Table A2:** Location of first distant recurrence in patients who received neoadjuvant chemotherapy.

	Total	DTC-Positive *n* (%)	*p*-Value	≥2 DTCs/1.5 × 10^6^ Cells *n* (%)	*p*-Value
Bone only					
yes	3	1		0	
no	18	11	0.386	5	-
Visceral					
yes	16	9		4	
no	5	2	0.525	1	0.816

DTC = disseminated tumor cells.

**Table A3:** Location of first distant recurrence in patients who received adjuvant chemotherapy.

	Total	DTC-Positive *n* (%)	*p*-Value	≥2 DTCs/1.5 × 10^6^ Cells *n* (%)	*p*-Value
Bone only					
yes	14	4		1	
no	39	9	0.685	0	0.099
Visceral					
yes	27	7		0	
no	26	6	0.806	1	0.229

DTC = disseminated tumor cells.
